# Design and Parameter Optimization of Quasi-Zero-Stiffness Structures Based on Cosine-Curve Compliant Beams

**DOI:** 10.3390/mi16080961

**Published:** 2025-08-21

**Authors:** Zhuo Sun, Jinpeng Hu, Deng Li, Long Huang

**Affiliations:** 1 College of Mechanical and Vehicle Engineering, Changsha University of Science and Technology, Changsha 410114, China; szhuo12@163.com; 2 Hunan Provincial Key Laboratory of Intelligent Manufacturing Technology for High-Performance Mechanical Equipment, Changsha University of Science and Technology, Changsha 410114, China; 15798090198@163.com; 3 College of Mechanical and Energy Engineering, Beijing University of Technology, Beijing 100124, China; lideng@emails.bjut.edu.cn

**Keywords:** quasi-zero-stiffness, cosine-curve beam, compliant structure, parameter optimization

## Abstract

Quasi-zero-stiffness (QZS) structures can provide a near constant force output in a certain range of displacement without force sensors and controllers. Therefore, they can be used in overload protection, vibration isolation, and biomedical application. In this paper, we propose a novel QZS structure based on cosine-curve compliant beams, which have a large QZS stoke and compact layout. The proposed QZS structure is composed of two half-period cosine-curve compliant beams with negative stiffness and two one-period cosine-curve compliant beams with positive stiffness. Then, we conducted the modeling of the force-displacement relationship of the compliant beams and analyzed the influence of the parameters on the mechanical performance. Based on the influence analysis, we propose the optimization processes to achieve QZS and obtain a QZS structure with the required force-displacement behavior. Finally, the mechanical performance of the QZS structure is verified through compression experiments on the prototype.

## 1. Introduction

Unlike linear elastic structures, quasi-zero-stiffness (QZS) structures are capable of maintaining constant force in a certain range of displacement without force sensors and controllers, and therefore they are widely used in scenarios such as compliant gripping [1,2,3,4,5], passive vibration isolation [6,7,8,9,10,11], energy absorption [12,13,14] and mechanical metamaterials [15,16,17,18].

A conventional approach for constructing QZS structures is to combine negative and positive stiffness modules. In the field of quasi-zero-stiffness vibration isolators based on rigid structures, Carrella et al. constructed the foundational model by integrating three springs. A pair of inclined springs provided negative stiffness, while an upright linear spring contributed positive stiffness; the interaction of these components resulted in the quasi-zero-stiffness characteristic [19]. Liu et al. proposed a quasi-zero-stiffness vibration isolator based on a nonlinear negative stiffness configuration. This system is composed of four vertical springs and eight symmetrically arranged pre-compressed helical springs, where the vertical spring system provided positive stiffness and the pre-compressed helical spring system generated negative stiffness, enabling effective stiffness cancellation [20]. Yang et al. introduced a novel quasi-zero-stiffness nonlinear low-frequency vibration isolator utilizing a symmetrical connecting rod structure. By optimizing the length ratio of the connecting rods, they effectively extended the operational range of quasi-zero-stiffness, thereby enhancing the isolator’s load-bearing capacity [21]. Zhou et al. developed a quasi-zero-stiffness vibration isolator integrating a cam-roller-spring mechanism. This design demonstrated excellent vibration isolation performance under relatively small excitation amplitudes. With the increase in the excitation amplitude, the vibration isolation performance is further enhanced, effectively reducing the initial vibration isolation frequency and improving the overall vibration isolation efficiency [22]. Wang et al. proposed a new double-four-bar horizontal large-amplitude quasi-zero-stiffness vibration isolator based on the singular configuration and gravity compensation properties of a planar four-bar mechanism. Compared with conventional spring-based vibration isolators, this design offers advantages in structural simplicity and compactness, and demonstrates good vibration isolation performance under low-frequency and large-amplitude external excitations [23]. Zou et al. proposed a device for customizable nonlinear forces, which consists of a custom raceway, a pre-compressed spring, and a rolling bearing. It has achieved precise control of multiple nonlinear mechanical properties. Moreover, it has been verified by numerical simulation and experiments, and can be widely applied to energy harvesting, vibration isolation, and nonlinear energy sink, etc. [24].

The nonlinear elastic property of the QZS structure can be realized by using obliquely mounted springs and specially shaped compliant mechanisms. In comparison with the QZS structure based on obliquely mounted springs, the QZS structure based on compliant mechanisms has the advantages of a compact structure and no friction, and they are more suitable for application in precision instruments. Chen et al. considered the adaptive needs of the robot end effector and superimposed the negative stiffness of the multi-stable mechanism and the positive stiffness of the linear spring to construct a QZS mechanism [25]. Fan et al. developed a vibration isolation metamaterial composed of numerous unit cells exhibiting QZS property, which integrates the snap-through behavior of a sinusoidal beam and bending-dominated support of two semicircular arches, and reasonably designed the parameters of unit cells [26]. Winterflood et al. attained QZS through the integration of two Euler buckling beams oriented in opposing directions; one Euler buckling beam has negative stiffness, and another Euler buckling beam has positive stiffness [27]. Sui et al. proposed a QZS structure based on a compliant inclined trapezoidal beam and researched the nonlinear stiffness of an inclined trapezoidal beam influenced by buckling effects through finite element analysis. Additionally, paralleling linear positive stiffness springs to create QZS isolators [28]. Ding et al. developed a rigid-flexible coupling constant-force structure by combining a diamond-shaped mechanism and a nonlinear bi-stable beam [29]. Yu et al. designed a QZS compliant vibration isolator that is symmetrically arranged along the horizontal axis based on the principle of Roberts configuration; the positive module is based on a parallelogram compliant mechanism, while the negative module consists of four inclined fixed-guided compliant beams [30]. Zhou et al. proposed a QZS system under a variable load composed of air springs with positive stiffness and Euler buckled beams with negative stiffness, and verified static mechanical characteristics of the system through finite element analysis [31]. Zhang et al. developed a torsional and compliant QZS structure composed of three piecewise straight beams and three curved beams, which acted as a positive stiffness module and a negative stiffness module, respectively [32]. Huang et al. developed a QZS vibration isolator by parallel connecting a negative stiffness corrector, which used compressed Euler beams to a linear isolator [33]. Aiming at the compact structure requirement in application, Chen et al. developed a QZS joint by setting two sets of opposed compressed leaf springs in the horizontal direction as a buckling Euler beam structure, and the movement of the slider is used to change the spring effective length and thus the stiffness of the springs in the horizontal and vertical directions [34]. Zhou et al. designed a QZS vibration isolator with double-arc flexible beams (D-AFB) as array units, which provides an important reference for the development of miniaturization and integration [35]. Liu et al. developed a QZS metamaterial with a long bandwidth and compact structure by combining a positive stiffness heart-shaped beam and a negative stiffness cosine beam into a single cell, and applied it to underwater gliders to realize ultra-low frequency vibration isolation [36]. Huo et al. developed a new mechanical metamaterial that arranges QZS units in a Cartesian pattern using curved beams. The team initially modeled the QZS unit based on an Euler beam with a cosine configuration, thus creating periodic QZS metastructures with both horizontal and vertical patterns [37].

Another practical approach for constructing QZS structures is to optimize the shape of the structure and achieve QZS directly. In comparison with the first approach, this approach has the advantage of a large design space and compact structure. However, the complex shape of the structure may lead to stress concentration. Miao et al. proposed a distributed shape optimization method to design a QZS compliant clamping mechanism, which can achieve constant force output within a certain displacement input range [38]. Zhang et al. optimized the shape of the slender beam through the representation of the beam with a non-uniform rational B-spline with six control points [39]. Lin et al. used the shape function as a polynomial to optimize the shape and size of the folded flexible beam, and designed a new two-dimensional vibration isolation metamaterial with QZS characteristics in both horizontal and vertical directions [40]. Zhang et al. developed a compliant constant-force mechanism utilizing the second bending mode of fixed-guided compliant beams and designed a prototype providing a constant contact force of 40 N in robotic polishing [41]. Tong et al. designed a QZS constant-force mechanism that combined a bistable compliant beam for negative stiffness with a U-shaped structure for positive stiffness and proposed an automatic optimization method for a compliant constant-force mechanism based on finite element analysis and multi-objective genetic algorithm methods [42]. Xu et al. designed a compact W-shaped compliant mechanism with direct QZS and used the integrated compliant mechanism to achieve convenience without assembly and the miniaturization of the mechanism [43]. Hou et al. proposed a four-point constraint method to design the monolithic compliant beams with cubic spline characteristics, thus developing a QZS mechanism consisting of monolithic compliant curved beams [44]. Inspired by the petiole of a palm leaf, Feng et al. presented a novel nonlinear antivibration ring with deformable crescent-shaped cross-sections, which has significant nonlinear QZS characteristics [45].

In this paper, we propose a novel QZS structure based on cosine curve compliant beams. The proposed structure has a large QZS stoke and compact layout. In Section 2, the geometrical description of the cosine curve compliant beams is presented, and the equilibrium configuration for the compliant beams is derived by combining the minimum potential energy principle and optimal control method. In Section 3, we studied and analyzed the influence of the parameters of positive and negative stiffness cosine beams on their force and displacement curves. In Section 4, an optimal design method for QZS structures is proposed. Through parameter optimization of cosine beams with different periods, a quasi-zero-stiffness structure that meets the needs of different scenarios is obtained based on the proposed approach. In Section 5, the static characteristics of the QZS structures under compression are verified through experiments and simulations.

## 2. Design of the QZS Structure Based on Compliant Curved Beams

This paper proposes a novel QZS structure based on compliant curved beams with negative-stiffness and positive-stiffness. The backbone shape of the negative-stiffness beam is a cosine curve within half of the period, while the backbone shape of the positive-stiffness beam is a cosine curve within a period; they are defined by the following equations:

(1) $$y_{1}(x)=\frac{h_{1}}{2}\left[1-\cos(\frac{\pi x}{L_{1}})\right]\begin{array}{cc} & x\in \left[0,L_{1}\right] \end{array}$$

(2) $$y_{2}(x)=\frac{h_{2}}{2}\left[1-\cos(\frac{2\pi x}{L_{2}})\right]\begin{array}{cc} & x\in \left[0,L_{2}\right] \end{array}$$

where *L*_1_ and *L*_2_ denote the length of the curves on the *x*-axis, and *h*_1_ and *h*_2_ denote the peak-to-peak amplitude of the curves.

**Figure 1:**
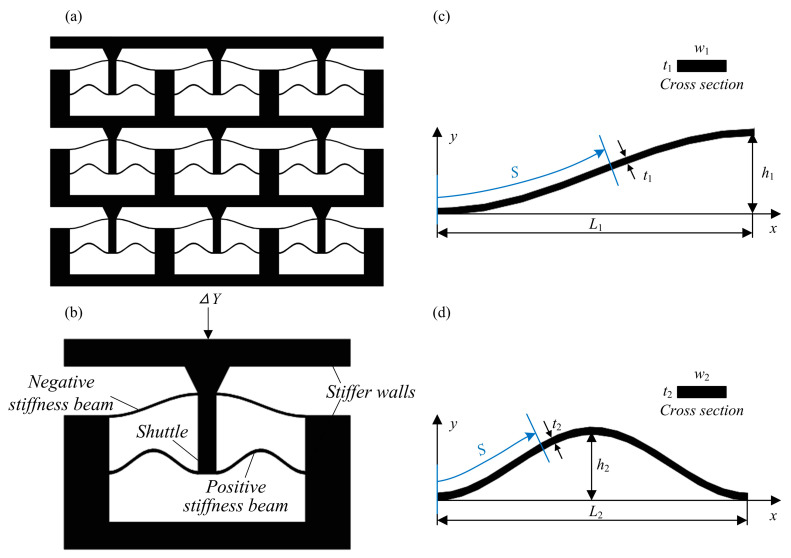
(**a**) Structural model, (**b**) unit cell, (**c**) the curves of the half-period cosine-curve beam; and (**d**) the curves of one-period cosine-curve beam.

Four cosine-curve beams are used to support the central shuttle symmetrically, as shown in Figure 1. This symmetric arrangement ensures that the force and displacement characteristics of the mechanism are balanced in different directions of the Y-axis translation, that is, it confines the degrees of freedom for the tips of the cosine-curve beam except in the Y-direction. The tips of these cosine-curve beams are connected to the shuttle, and through this connection, the deformation of the beams is transferred to the shuttle’s motion. The other ends of the cosine-curve beams are fixed in the solid frame with high stiffness. This rigid frame serves as a stable base for the entire QZS structure. The key role of this frame-beam-shuttle setup is to confine the degrees of freedom of the shuttle-connected beam tips strictly to the Y-direction: during the operation, when external forces act on the shuttle, the beams deform elastically, but the frame restricts non-Y-direction displacements of the beam ends. When the shuttle translates in the Y-axis direction, the compliant mechanism is expected to output a constant force in a certain range.

As shown in Figure 1, the characteristics of the proposed QZS structure can be derived from its unit cell. The number of units in the X-direction determines the load capacity of the structure, while the number of units in the Y-direction determines the displacement range of zero-stiffness. The proposed QZS structure can be customized to adapt to different applications. Here, the static mechanics of the QZS unit cell is modeled utilizing the energy approach.

According to the principle of minimum potential energy [46,47,48,49], under the given displacement boundary conditions, the stable equilibrium configuration of the elastic system corresponds to a local minimum of the potential energy. Therefore, we control the curvature function to minimize the total potential energy of the beam, thereby obtaining the deformation and end reaction force of the compliant beam. Here, we adopt the Frente-frame along the arc-length description and the Euler-Bernoulli beam theory to model each curved beam [50], and then we obtain the potential energy of a curved beam as

(3) $$J_{beam}=\int \frac{1}{2}EI{\left(\omega (s)-\omega^{*}(s)\right)}^{2}ds$$

where *E* and *I* represent Young’s modulus and the moment of inertia of the cross-section. *EI* is the product of the *E* and *I*, representing the bending stiffness of the component; *ɷ* and ɷ* represent the backbone curvature of the beam before and after deformation; *s* represents the arc length from the initial end to the end of the compliant beams. Then, the potential energy of the compliant mechanism is obtained as

(4) $$J_{mech}=\int EI{\left(\omega_{p}(s)-\omega_{p}^{*}(s)\right)}^{2}ds+\int EI{\left(\omega_{n}(s)-\omega_{n}^{*}(s)\right)}^{2}ds$$

where subscripts *p* and *n* denote the positive-stiffness beam and negative-stiffness beam.

The larger the values of *w* and *t* are, the greater *EI* will be, so the constant force *F* corresponding to the same Δ*Y* will be greater. Based on the above equation, we can use optimal control methods to minimize the potential energy for an arbitrary given tip-displacement input and obtain the equilibrium configuration for this condition. In this paper, we utilize the pseudo-spectral method to transform the optimal control problem into a nonlinear programming problem, which can be solved by using the fmincon function in MATLAB R2024b [51,52]. The basic principle of the pseudo-spectral method is as follows: At the forward mating point, the continuous optimal control problem is discretized. The state and control variables are approximately represented by global interpolation polynomials. The derivatives of the state variables in the dynamic equation with respect to time are approximated by differentiating the polynomials, and the function constraints of the equation are satisfied at the forward mating point. Thus, the optimal control problem is transformed into a nonlinear programming problem and then solved by methods such as the interior point method.

## 3. Parametric Analysis of the Cosine-Curve Beam

**Figure 2:**
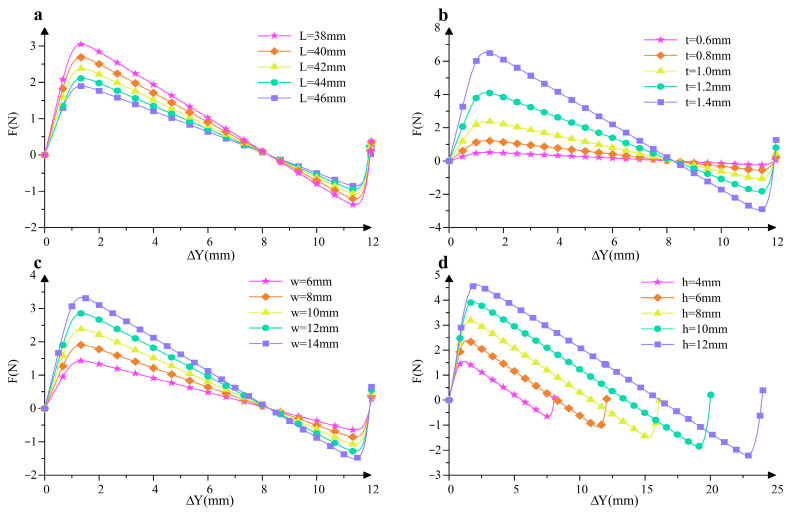
Force-displacement curves of the half-period cosine-curve beam with different parameters: (**a**) different *L*_1_; (**b**) different *t*_1_; (**c**) different *w*_1_; and (**d**) different *h*_1_.

**Figure 3:**
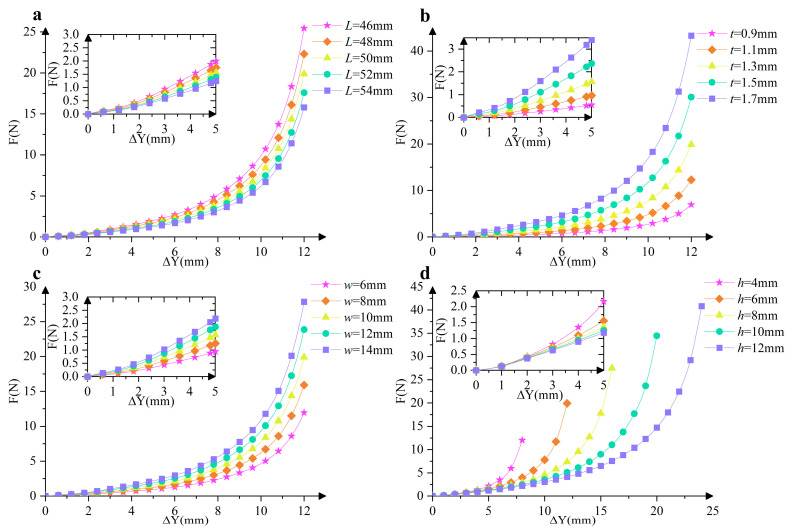
Force-displacement curves of the one-period cosine-curve beam with different parameters: (**a**) different *L*_2_; (**b**) different *t*_2_; (**c**) different *w*_2_; and (**d**) different *h*_2_.

Through the above approach, we obtain the mechanics of the cosine-curve beam for different parameter values. Except for the period, the mechanical property of the cosine-curve beam is determined by the following parameters: the length of the curve in the *x*-axis (*L*), the width (*w*), the thickness (*t*), and the amplitude of the curve (*h*). For the convenience of comparing the influences of the parameters on the mechanical property, here the standard parameters are determined: for half-period cosine-curve beams, *L*_1_ = 50 mm, *w*_1_ = 10 mm, *t*_1_ = 1 mm, *h*_1_ = 6 mm; for one-period cosine-curve beams, *L*_2_ = 50 mm, *w*_2_ = 10 mm, *t*_2_ = 1.3 mm, *h*_2_ = 6 mm. It should be specifically noted that Δ*Y* represents the vertical displacement of the unit cell when it transitions from the initial natural state to the compressed state, and this definition is clearly illustrated in Figure 1b. It is important to clarify that there is no explicit analytical formula that can directly describe their connection between force *F* and Δ*Y*. Instead, this relationship is derived through a rigorous numerical optimization process based on Equation (4), which involves iteratively adjusting variables to minimize the error between the calculated and target values, thereby obtaining the corresponding force values for different vertical displacements. By applying the proposed method to the inverse problem of these two kinds of beams, respectively, we obtain the force-displacement curve for different parameters, as shown in Figure 2 and Figure 3.

The *F-*Δ*Y* curves in these figures not only visually exhibit how the force changes with the vertical displacement under different parameter combinations but also serve as a key tool to quantitatively analyze the effects of varying parameters *L*, *t*, *w*, and *h* on QZS characteristics. Moreover, these curves provide a solid foundation for determining the direction of parameter adjustment in the optimization of QZS mechanisms, allowing us to identify which parameters need to be increased or decreased to achieve the desired mechanical performance.

It can be observed that the half-period cosine-curve beam presents an obvious three-stage stiffness feature, and the negative stiffness almost remains constant within a certain range of the shuttle’s displacement. For the half-period cosine-curve beam, if the other parameters are given, the absolute value of the stiffness slowly decreases with the increase in *L*. The width and thickness are two key parameters for the moment of inertia of the cross-section, and therefore, the absolute value of the stiffness increases with the increase in *w* and *t* at different rates; also, they have no influence on the variation trend and the location of the equilibrium configuration. If the amplitude of the curve increases, the absolute value of the negative stiffness almost remains constant, while the displacement range corresponds to negative stiffness increases. As shown in Figure 3, the one-period cosine-curve beam presents a two-stage positive stiffness feature: the stiffness in the first stage almost remains constant, and it is significantly smaller than the stiffness in the second stage. If the other parameters are given, the positive stiffness slowly decreases with the increase in *L*. Similarly, as the width and thickness increase, the moment of inertia of the cross-section increases, and the stiffness also increases. It is noteworthy that the excessive increase in thickness may result in the failure of the Euler–Bernoulli beam assumption and the linear elastic assumption. In addition, if *h* increases, the positive stiffness in the first stage decreases very slowly, while the displacement range of the first stage increases. In comparison with *L* and *w*, parameters *t* and *h* have more obvious influences on the stiffness and the maximum value of the equilibrium force. Therefore, during the optimal design processes of the constant-force mechanism, parameters *L* and *w* for both cosine-curve beams are pre-determined (*L* = 50 mm, *w* = 10 mm), while *t* and *h* are selected as two key parameters to be optimized.

## 4. Parameter Optimization of QZS Structures

**Figure 4:**
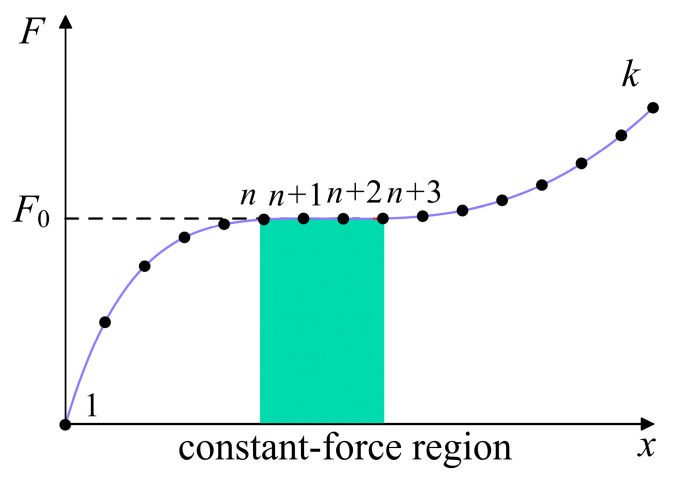
Discretization of the force-displacement curve. The shaded region is the constant-force region.

Before the optimization of the compliant mechanism, we conduct the discretization for the force-displacement curve according to Refs. [53,54]. First, the force-displacement curve of the mechanism is discretized with the *y*-axis displacement uniformly divided, as shown in Figure 4. Once the required constant-force region is determined, the corresponding discrete points for the region can be obtained, which are denoted by *n*, …, *n* + k. If the corresponding forces (F (*n*), …, F (*n* + k)) approximate each other, then the mechanism is considered a constant-force mechanism in this region. Considering precision and calculation complexity, here we choose four discrete points for the constant-force region. In addition, to improve efficiency, the parameters *h* and *t* can be confined according to the workspace of the mechanism and the yield limit of the material.

For the selected discrete points, the objective function is defined as

(5) $$F_{obj}=\left[\left|F(n)-F(n+1)\right|+\left|F(n+1)-F(n+2)\right|+\left|F(n+2)-F(n+3)\right|+\left|F(n+3)-F(n)\right|\right]/4F(n)$$

which includes the force deviation between adjacent points and the force deviation between the first and last points.

**Figure 5:**
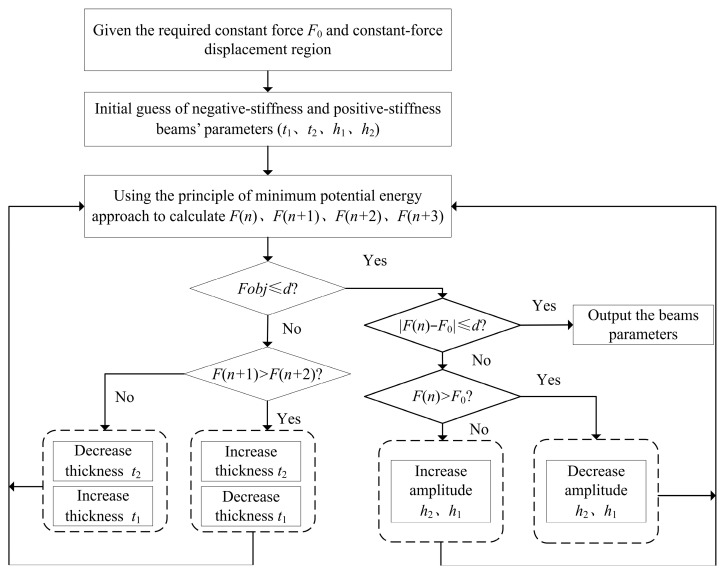
Parameter optimization processes for the QZS structures with cosine-curve beams.

Based on the above discretization, we propose the parameter optimization processes for the QZS structures with cosine-curve beams, as shown in Figure 5. First, substitute the initial guess of the parameters to the solving procedures of the proposed method, then we obtain F (*n*), F (*n* + 1), F (*n* + 2) and F (*n* + 3) in the required displacement range. Then, adjust parameters *t*_1_ and *t*_2_ accordingly, until the objective function is within the allowable errors *d*_1_, and we will obtain a constant-force mechanism in the required displacement range. Furthermore, compare the value of the constant force with the required force, and adjust parameters *t*_1_ and *t*_2_ accordingly, until the deviation ratio of the constant force is within the allowable errors *d*_2_, and we will obtain a constant-force mechanism outputting the required constant force in the required displacement range.

## 5. Simulation and Test

As a case study, here the required constant force *F*_0_ is set as 5.5 N, and the required displacement range is 2~5 mm; the allowable errors *d*_1_ and *d*_2_ are both set as 5%. The material of both cosine-curve beams is photosensitive resin. Tensile tests of the material show that its Young’s modulus is 1.402 GPa and almost remains constant within 4% strain. Considering the structural compactness and linear elastic limit, the following constraints are applied to the parameters.

(6) $$\left\{\begin{array}{c} 1\mathrm{mm}<h_{1}<5\mathrm{mm}\\ 1\mathrm{mm}<h_{2}<5\mathrm{mm}\\ 0.2\mathrm{mm}<t_{1}<1\mathrm{mm}\\ 0.2\mathrm{mm}<t_{2}<1\mathrm{mm} \end{array}\right.$$

As a result of the optimization processes, the parameters for the cosine-curve beams are shown in Table 1. In the displacement range of 1~5 mm, the corresponding theoretical tip force is in the range of 5.469~5.660 N, which is within the ±2.90% range of the required constant force.

**Figure 6:**
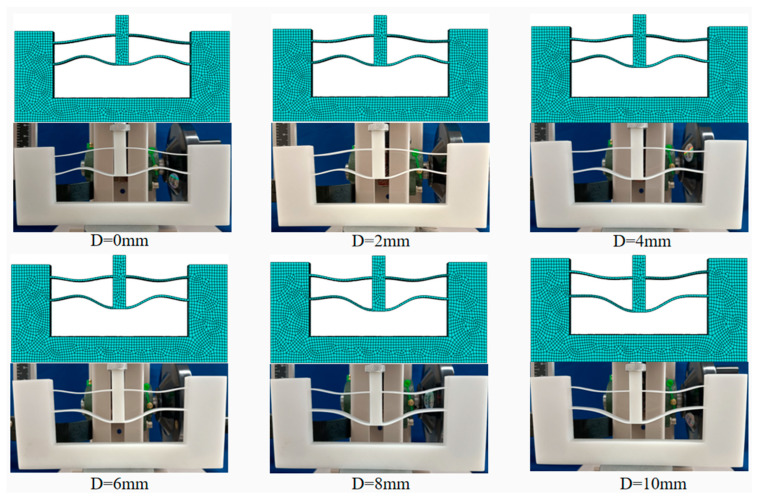
Equilibrium configurations for different shuttle displacements in simulation and tests.

**Figure 7:**
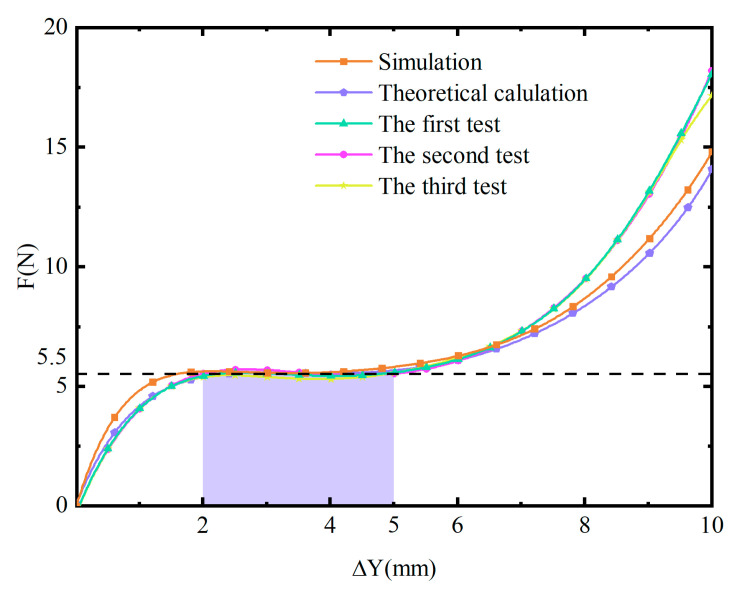
Force-displacement curves obtained through theoretical calculation, simulation, and tests. The shaded region is regarded as the constant-force region.

To verify the mechanical properties of the above resultant mechanism, we conducted simulations on the finite element model in Abaqus 2022 software. The mechanical properties of the photosensitive resin were incorporated into the material settings of the software. The material is assumed to be isotropic and homogeneous. Each unit is meshed by C3D4 elements with a mesh size of 1 mm. During the simulation, the bottom surface and both side surfaces of the solid frame were fixed. The displacement is applied on the top surface of the shuttle with 0.5 mm/s in velocity, and the equilibrium configurations for different displacements were obtained, as shown in Figure 6. Furthermore, we conducted compression tests on 3D-printed prototypes made from photosensitive resin. The Young’s modulus of the photosensitive resin is 0.8 GPa. The test machine was set in the displacement-controlled mode with 0.5 mm/s a velocity. Then, we obtained the corresponding equilibrium configurations, which were also shown in Figure 7. It can be observed that the deformations of the beams in the simulation and test match very well. Figure 7 shows the force-displacement curves obtained through theoretical calculation, simulation, and tests. During the displacement range of 2~5 mm, the forces on the shuttle obtained through simulation and tests are 5.309~5.706 N and 5.552~5.740 N, respectively, whose deviation ratios with regard to F0 are ±3.74% and ±4.36%. The deviation of the test results is slightly larger than that of the simulation and theoretical results due to the printing error of the prototype.

**Figure 8:**
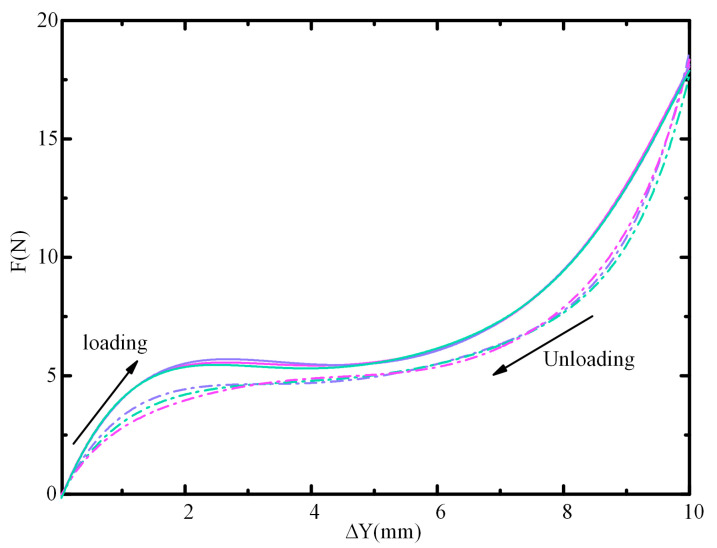
Force–displacement curves during reduplicate loading and unloading tests.

In addition, to verify the stability of the constant force, we also conducted reduplicate loading and unloading tests. The obtained force-displacement curves are shown in Figure 8. During the reduplicate loading tests, the force exerted on the shuttle for the displacement range of 2~5 mm is approximately the required force; the deviation ratios are within 2.1%. During the unloading tests, the force–displacement curves demonstrate a smaller constant force, which is probably induced by the material hysteresis.

## 6. Conclusions

In this paper, we designed a new QZS structure and demonstrated its static characteristics through theoretical and experimental analyses. First, based on the principle of minimum potential energy, we designed a QZS structure composed of positive and negative stiffness cosine beams. Then, we construct a novel type of QZS structure through the combination of two types of cosine-curve beams. The property of the structures is verified through simulation and tests. The resultant QZS structure has the advantages of structural compactness and extensibility, which can be applied to the compliant gripper and the polishing tool with a large workspace. Furthermore, it can be easily used to construct a QZS metamaterial with low-frequency vibration isolation properties.

## Figures and Tables

**Table 1:** Parameters of the compliant mechanism after optimization.

Parameters of the Half-Period Cosine-Curve Beam				Parameters of the Half-Period Cosine-Curve Beam			
*L*_1_ (mm)	*h*_1_ (mm)	*w*_1_ (mm)	*t*_1_ (mm)	*L*_2_ (mm)	*h*_2_ (mm)	*w*_2_ (mm)	*t*_2_ (mm)
20	6	5	0.4	20	3	5	0.5

## Data Availability

The original contributions presented in this study are included in the article. Further inquiries can be directed at the corresponding authors.
